# Age-Related Changes in the Gut Microbiota Modify Brain Lipid Composition

**DOI:** 10.3389/fcimb.2019.00444

**Published:** 2020-01-14

**Authors:** Mayssa Albouery, Bénédicte Buteau, Stéphane Grégoire, Claire Cherbuy, Jean-Paul Pais de Barros, Lucy Martine, Florian Chain, Stéphanie Cabaret, Olivier Berdeaux, Alain M. Bron, Niyazi Acar, Philippe Langella, Marie-Agnès Bringer

**Affiliations:** ^1^Centre des Sciences du Goût et de l'Alimentation, AgroSup Dijon, CNRS, INRAE, University of Bourgogne Franche-Comté, Eye and Nutrition Research Group, Dijon, France; ^2^Micalis Institute, INRAE, AgroParisTech, University Paris-Saclay, Jouy-en-Josas, France; ^3^Inserm U1231 “Lipids, Nutrition, Cancer”, Lipidomic Platform, University of Bourgogne Franche-Comté, Dijon, France; ^4^Centre des Sciences du Goût et de l'Alimentation, AgroSup Dijon, CNRS, INRAE, University of Bourgogne Franche-Comté, ChemoSens Platform, Dijon, France; ^5^Department of Ophthalmology, University Hospital, Dijon, France

**Keywords:** aging, microbiota, lipid, cholesterol, fatty acid, phospholipid, liver, cortex

## Abstract

Understanding the molecular mechanisms underlying the changes observed during aging is a prerequisite to design strategies to prevent age-related diseases. Aging is associated with metabolic changes, including alteration in the brain lipid metabolism. These alterations may contribute to the development of pathophysiological conditions. Modifications in the gut microbiota composition are also observed during aging. As communication axes exist between the gut microbiota and the brain and knowing that microbiota influences the host metabolism, we speculated on whether age-associated modifications in the gut microbiota could be involved in the lipid changes observed in aging brain. For that purpose, germ-free mice were colonized by the fecal microbiota of young or old donor mice. Lipid classes and fatty acid profiles were determined in the brain (cortex), plasma and liver by thin-layer chromatography on silica gel-coated quartz rods and gas chromatography. Gut colonization by microbiota of old mice resulted in a significant increase in total monounsaturated fatty acids (MUFA) and a significant decrease in the relative amounts of cholesterol and total polyunsaturated fatty acids (PUFA) in the cortex. Among the eight most represented fatty acids in the cortex, the relative abundances of five (C18:1n-9, C22:6n-3, C20:4n-6, C18:1n-7, and C20:1n-9) were significantly altered in mice inoculated with an aged microbiota. Liquid chromatography analyses revealed that the relative abundance of major species among phosphatidyl and plasmenylcholine (PC 16:0/18:1), phosphatidyl and plasmenylethanolamine (PE 18:0/22:6), lysophosphatidylethanolamine (LPE 22:6) and sphingomyelins (SM d18:1/18:0) were significantly altered in the cortex of mice colonized by the microbiota obtained from aged donors. Transplantation of microbiota from old mice also modified the lipid class and fatty acid content in the liver. Finally, we found that the expression of several genes involved in MUFA and PUFA synthesis (*Scd1, Fads1, Fads2, Elovl2*, and *Elovl5*) was dysregulated in mice inoculated with an aged microbiota. In conclusion, our data suggest that changes in gut microbiota that are associated with aging can impact brain and liver lipid metabolisms. Lipid changes induced by an aged microbiota recapitulate some features of aging, thus pointing out the potential role of microbiota alterations in the age-related degradation of the health status.

## Introduction

Aging leads to cell degeneration and deterioration of organ structure and function on an irreversible and gradual manner. These conditions make aging a primary risk factor for major human disorders including cancers, metabolic and neurodegenerative diseases (Kennedy et al., 2014; Zierer et al., 2015). Aging is a complex process that involves both, genetic and environmental factors, notably changes in the gut microbiota that is getting more attention.

The term gut microbiota refers to the complex microbial communities that consist of bacteria, fungi, archea and viruses that inhabit the gastrointestinal tract. The resident gut microbiota and its host maintain a mutually beneficial relationship. A healthy host offers a stable environment and nutrients to the microbiota. In turn, by producing bioactive metabolites, gut microbes contribute to maintain homeostasis and ensure healthiness for the host. At the gut level, microbiota fulfills several functions that include intestinal development as well as immunomodulatory, metabolic and protective functions (Jandhyala et al., 2015; Adak and Khan, 2019). It also influences the physiological functioning of distant organs through several routes including neural, endocrine, immune, and metabolic pathways. This is particularly well documented regarding the gut-brain axis (Dinan and Cryan, 2017; Ma et al., 2019). Although it is relatively stable throughout adult-life, the composition of the gut microbiota changes in elderly (Lozupone et al., 2012; O'Toole and Jeffery, 2018). These modifications (gain or loss of community members or changes in their relative abundance), termed dysbiosis, can impact the metabolic activity of the microbiota, with consequences on its mutualistic relationship with the host. Indeed, microbiota dysbiosis is associated with the pathogenesis of age-related diseases, including metabolic diseases, as well as cancers, neurodegenerative and psychiatric disorders (Alvarez-Mercado et al., 2019; Ding et al., 2019).

Lipid homeostasis is crucial for host physiology and health. Indeed, lipids are essential components of biological membranes, they constitute energy sources and, as being signaling molecules, they also take part in the regulation of cell metabolism and homeostasis. Changes in lipid metabolism have been reported during aging and in age-related diseases, and the light has been shed on connections between lipid composition of tissues and longevity in human and model organisms (Rappley et al., 2009; Schroeder and Brunet, 2015; de Diego et al., 2019). The intestinal microbiota plays a critical role in protein, carbohydrate and lipid metabolism. Evidence coming from comparisons between germ-free and conventionally raised rodents indicates that the presence of microbes in the gut has an impact on the lipid composition of metabolically important organs such as adipose tissue and liver, but also lipid-rich organs such as the retina (Backhed et al., 2004, 2007; Oresic et al., 2009; Rabot et al., 2010; Velagapudi et al., 2010). The composition of the gut microbiota also influences lipids in organs. Microbial dysbiosis are associated with metabolic diseases such as obesity and diabetes, and the use of prebiotics or probiotic bacteria was shown to ameliorate metabolic syndrome at least by acting on lipid metabolism (Yang and Kweon, 2016; He and Shi, 2017). Regulation of the lipid metabolism by gut microbes takes place at the gut level but also in distant organs. The microbiota regulates lipid metabolization and absorption at the level of the intestinal mucosa (Martinez-Guryn et al., 2018). In addition to that, gut microbes can modify hepatic *de novo* lipogenesis by modulating expression of genes involved in lipid metabolism and by generating short chain fatty acids (SCFA) that are substrates for hepatic lipogenesis (Singh et al., 2015; Kindt et al., 2018).

Within human tissues, brain is one displaying the highest content in lipids, and diversity of lipid classes and molecular species (Han, 2007). Modifications of the brain lipid composition have been described in aged healthy and unhealthy brain (Rappley et al., 2009; Naudi et al., 2015). Considering the crucial roles played by gut microbes in the regulation of lipid metabolism and the existence of a gut-brain axis, we sought to evaluate whether changes occurring in the gut microbiota during aging would impact brain lipids. We inoculated germ-free mice with the microbiota of young or old conventionally raised mice. We found that animals colonized with an aged microbiota displayed alterations of lipid profiles in the cortex and the liver. They were characterized by a decrease in the cholesterol and polyunsaturated fatty acids (PUFA) levels, while the monounsaturated fatty acid (MUFA) levels were increased. Moreover, we showed that changes in the gut microbiota that are associated with aging induce the generation of MUFA and has an impact on PUFA elongation and unsaturation, by modifying the expression levels of several genes (*Scd1, Fads1, Fads2, Elovl2*, and *Elovl5*) encoding enzymes involved in fatty acid biosynthesis.

## Materials and Methods

### Animals

Germ-free C3H/HeN males were raised under germ-free conditions in isolators in the germ-free animal facility at the Anaxem platform. The bedding was sterile wood shavings. Mice were given free access to autoclaved tap water and a γ-irradiated (45 kGy) standard diet (R03; Scientific Animal Food and Engineering, Augy, France).

### Microbiota Transplantation

At 8 weeks of age, germ-free C3H/HeN mice were moved to two experimental isolators for fecal microbiota transplantation (1 isolator for mice that received the microbiota of young mice (*n* = 7) and 1 isolator for mice that received the microbiota of old mice (*n* = 8). Germ-free C3H/HeN mice were inoculated with 200 μL of fresh fecal sample in PBS solution of young or old mice, by oral gavage. The fecal microbiota of young mice was obtained from a pool of feces of three C57Bl/6JRj mice at 4 weeks of age (Janvier Labs, Le Genest-Saint-Isle, France). The fecal microbiota of old mice was obtained from feces of three C57Bl/6JRj mice at 24 months of age (Janvier Labs). Collected feces were weighted and diluted in sterile phosphate buffer saline (100 mg of feces/mL).

Mice were kept in experimental isolators throughout the experiment and were sacrificed 60 days after inoculation.

### Fecal Microbiota Analysis by 16S rRNA Gene Sequencing

All the following experiments were performed by Biomnigene (www.biomnigene.fr, Besançon, France). DNA was extracted from about 90 mg of frozen mouse fecal samples using the E.Z.N.A Stool DNA kit (Omega Bio-tek, Norcross, GA, USA) following the manufacturer's instructions. The V3-V4 hyper-variable region of the 16S rRNA gene was amplified by using the following primers: forward primer CCTACGGGNGGCWGCAG and reverse primer GGACTACHVGGGTWTCTAAT, in which bases indexes were incorporated to perform multiplexing. The PCR reactions were performed using 10 ng of fecal DNA, 0.4 μM primers, 200 μM dNTP, and 1X of PrimeSTAR® Max DNA Polymerase (Takara Bio Europe, Saint-Germain-en-Laye, France). Amplifications were carried out using the following program: 1 cycle at 98°C for 5 min, followed by 37 cycles at 98°C for 10 s, 58°C for 15 s, 72°C for 5 s, and finishing with a step at 72°C for 1 min. The PCR products were analyzed on Qiaxcel Cartridge (QIAGEN, Courtaboeuf, France) to verify the amplification. For the preparation of the libraries, the concentration of the PCR products was determined by fluorometric assay on Qubit™ 4.0 and the samples were grouped in an equimolar manner. The pool of PCR products was then purified by electrophoresis on PippinHT using a 1.5% agarose cassette (Sage Sciences, Beverly, MA, USA). The sequencing of V3-V4 amplicons was carried out on MiSeq Illumina in 2X251 bp by using the Illumina MiSeq Reagent Kit v2 (500 cycles; Illumina, San Diego, CA, USA).

### 16S rDNA Gene Sequences and Statistical Analysis

Sequences were analyzed using the FROGS pipeline to obtain the OTU (Operational Taxonomic Units or phylotypes) abundance table. The successive steps involved de-noising and clustering of the sequences into OTUs using SWARM; chimera removal using VSEARCH; taxonomic affiliation for each OTU using both RDP Classifier and NCBI Blast+ on Silva132 16S. Statistical analyses were performed using “R” language and environment version 3.2.3. β-diversity (UniFrac and weighted UniFrac dissimilarity) measurement and analysis of the differences in OTUs between samples were performed using the add-on package “Phyloseq” (Mcmurdie and Holmes, 2013). Differences in the microbial communities between groups were evaluated using constrained analysis of principal coordinates and permutational multivariate ANOVA. Statistical differences between groups for individual OTU abundance was calculated using the Mann-Whitney test with Benjamini-Hochberg false discovery rate correction. Statistical significance was set at *p* < 0.05.

### Lipid Class Distributions

Total lipids were extracted from plasma, livers and brains following the Folch's procedure (Folch et al., 1957). The distribution of lipids into different classes [phospholipids (PL), triglycerides (TG), diglycerides (DG), free fatty acids (FFA), free cholesterol (Chol), and cholesteryl esters (CE)] was determined using a combination of thin-layer chromatography on silica gel-coated quartz rods and flame ionization detection (Iatroscan® system, Iatron, Tokyo, Japan), according to Ackman's technique (Acar et al., 2009). The values obtained for each compound were corrected according to their response factor using specific calibration curves. Data were reported as a percentage relative to total lipids in the sample (considered as 100%).

### Fatty Acid Composition

Total lipids were transmethylated using boron trifluoride in methanol according to Morrison and Smith (Morrison and Smith, 1964). Fatty acid methyl esters (FAMEs) were subsequently extracted with hexane and analyzed on a GC Trace 1310 (Thermo Scientific, Les Ulis, France) gas chromatograph (Palo Alto, CA, USA) using a CPSIL-88 column (100 ×0.25 mm i.d., film thickness 0.20 μm; Varian, Les Ulis, France) equipped with a flame ionization detector. Hydrogen was used as carrier gas (inlet pressure 210 kPa). The oven temperature was held at 60°C for 5 min, increased to 165°C at 15°C/min and held for 1 min, and then to 225°C at 2°C/min and finally held at 225°C for 17 min. The injector and the detector were maintained at 250°C. FAMEs were identified by comparison with commercial and synthetic standards. The data were processed using the ChromQuest software (Thermo Scientific) and reported as a percentage relative to total fatty acids (considered as 100%).

### Characterization of Phospholipid Species

The Orbitrap Fusion™ Tribrid mass spectrometer (Thermo Scientific, USA) was used for high-resolution analyses in order to identify phospholipid molecular species in mouse brains. This instrument was equipped with an EASY-MAX NG™ Ion Source (Heated Electrospray Ionization H-ESI) and was controlled by Xcalibur™ 4.1 software. Positive and negative ions were monitored alternatively by switching polarity approach with a spray voltage set to 3,500 V in positive and negative ion modes. The Orbitrap mass analyzer was employed to obtain all mass spectra in full scan mode with the normal mass range and a resolution set value m/z 240,000 amu. A dynamic exclusion filter was applied with an exclusion duration of 15 s and a mass tolerance of 5 ppm. For tandem mass spectrometry (MS/MS) analyses, data-dependent mode was used for the characterization of PL species. Precursor isolation was performed in the Quadrupole analyzer with an isolation window of m/z 1.6 amu. Higher-energy Collisional Dissociation (HCD) was employed for the fragmentation of PL with an optimized collisional energy of 30 eV and a stepped collision energy of 5%. The linear ion trap was used to acquire spectra for fragment ions in data-dependent mode. The Automatic Gain control Target was set to 2.104 with a maximum injection time of 50 ms. The identification of all PL species was performed, using the data of high accuracy and the information collected from fragmentation spectra, with the help of the LipidSearch™ software and the LIPID MAPS® database.

### Individual Species of Phospholipid Analysis

High performance liquid chromatography (HPLC) was performed on an Agilent 1,200 equipped with an autosampler, a binary pump and a column oven. During analysis, the column (ZorBAX EclipsePlus C18 −2.1 × 100 mm, 1.8 μm, Agilent Technologies, Les Ulis, France) was maintained at 55°C. The mobile phases consisted of water/methanol (60/40 v/v) 10 mM ammonium acetate, 1 mM acetic acid (A) and of IPA/methanol (90/10) 10 mM ammonium acetate, 1 mM acetic acid (B). The linear gradient was as follows: 40% B for 1 min, up to 95% B in 15 min and maintained at 95 % for 1 min. Column was equilibrated with 40% B for 3 min before each injection. The flow rate was 0.25 mL/min. The autosampler temperature was set at 5°C. One to five microliters of sample were injected onto the HPLC system.

The HPLC system was coupled on-line to a 6,460 triple quadrupole mass spectrometer (MS^2^) equipped with Jet Stream electrospray ionization source (Agilent Technologies). Source parameters were set-up as follows: temperature set at 325°C, nebulizer gas (nitrogen) pressure 20 psi and flow rate 10 L/min, sheath gas (nitrogen) flow and temperature 11 L/min and 300°C respectively. The capillary and Nozzle voltages were adjusted to 3,500 and 1,000 V, respectively. Quantitation of phosphatidylcholines was achieved in SRM positive mode ([M+1]+ → 184.1; fragmentor 160 V, Collision energy 20 V). Quantitation of phosphatidylethanolamines was achieved in SRM positive mode ([M+1]+ → [M-140]+; fragmentor 136 V, Collision energy 12 V). Quantitation of phosphatidylinositols was achieved in SRM negative mode ([M-1]- → 241 (inositol); fragmentor 150 V, Collision energy 30 V). Relative quantitation of phospholipids was performed according to the area under curve of each species calculated with MassHunter Workstation Software (version B09, Agilent Technologies).

### Gene Expression

Total RNA was isolated from livers and brains using TRIzol reagent (Invitrogen, Thermo Fisher Scientific, Illkirch-Graffenstaden, France). cDNA was synthesized using High capacity cDNA Reverse transcription kit (Applied Biosystems, Thermo Fisher Scientific). Real time PCR was performed using C1000 Thermal cycler (Bio-Rad, Marnes-la-Coquette, France) in a reaction mixture of cDNA, iTaq Universal SYBR Green Supermix (Bio-Rad) and mouse-specific oligonucleotides (Supplementary Table 1). Results were normalized to the housekeeping *Hprt* gene.

### Statistical Analyses

Data are presented as the mean ± standard error of the mean (SEM). Statistical analyses were performed with GraphPad Prism software (GraphPad Software Inc., San Diego, CA, USA). The non-parametric Mann and Whitney test was used to compare results between groups. The *p***-**values ≤ 0.05 were considered as statistically significant.

## Results

### Characterization of the Microbiota Implanted in Germ-Free Mice After Transfer of Fecal Microbiota Coming From Young and Aged Donor Mice

**Figure 1:**
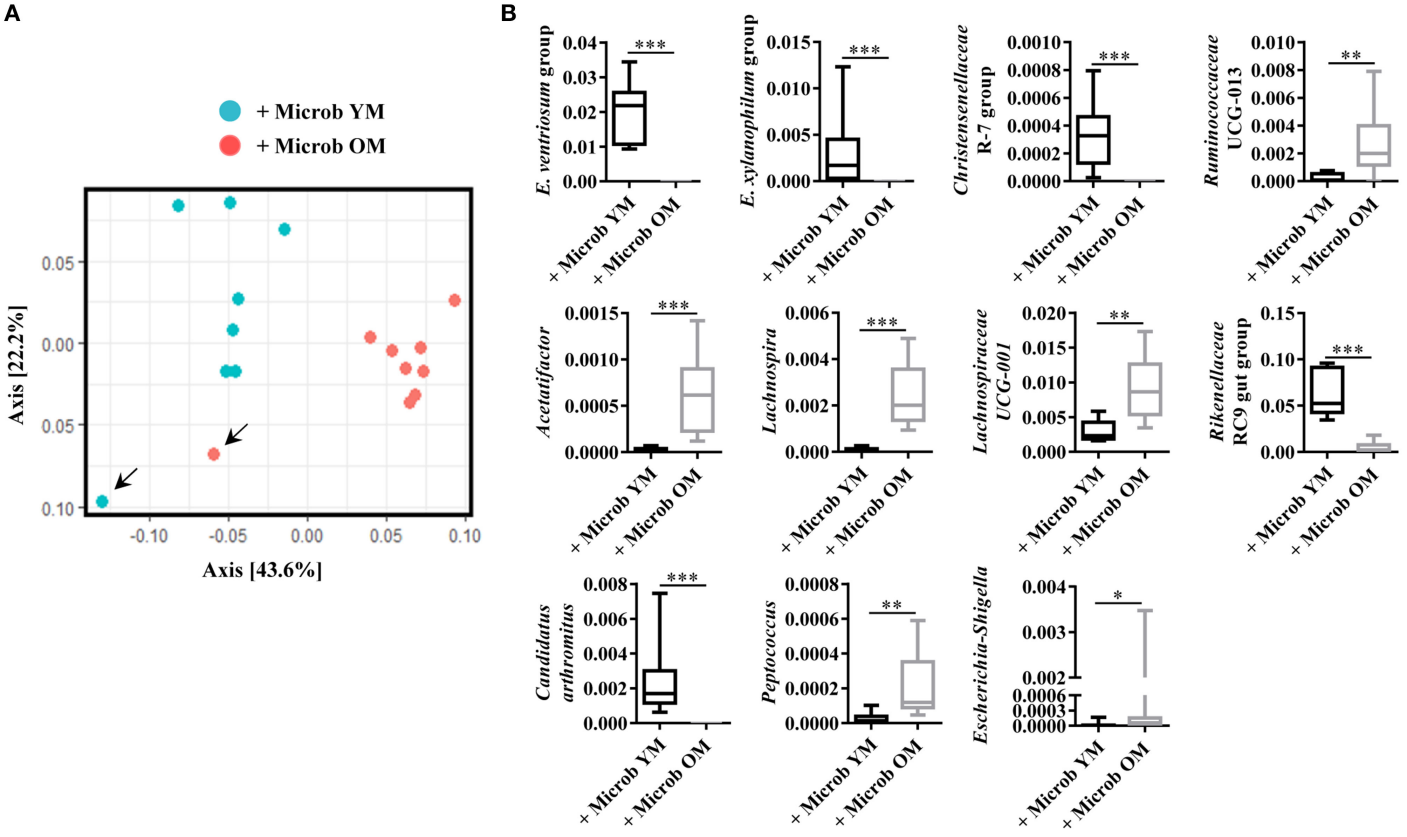
Microbiota analysis in recipient germ-free mice. **(A)** Composition of the fecal microbiota analyzed by Unifrac distance. Each dot represents one mouse. Blue dots represent fecal microbiota from germ-free mice (*n* = 7) inoculated with feces of conventionally raised young mice (+ Microb YM) and red dots represent fecal microbiota of germ-free mice (*n* = 8) inoculated with feces from conventionally raised old mice (+ Microb OM). Arrows indicate donors. **(B)** Relative abundance in percentage of genus significantly modified in fecal microbiota of mice inoculated with feces from young mice and of mice inoculated with feces from old mice. *E. ventriosum* group: [*Eubacterium*] *ventriosum* group, and *E. xylanophilum* group: [*Eubacterium*] *xylanophilum* group. Comparisons were done between the two groups of mice (+ Microb YM and + Microb OM). *p < 0.05, **p < 0.01 and ***p < 0.001.

Fecal samples freshly obtained from young or old mice were inoculated into germ-free recipient mice. Sixty days after the transfer, feces were recovered to characterize the microbiota. Based on Unifrac distance metrics, we observed that all the mice that have received microbiota from a same type of donors (young vs. old), clustered together (Figure 1A). This was confirmed with the constrained analysis of the principal coordinates and permutational multivariate ANOVA that showed significant differences between the 2 groups of animals (*p* < 0.001). Alpha-diversity was analyzed by calculating the Chao1 (estimated community richness) and Shannon's (estimated diversity that takes into account both richness and evenness) index. No significant differences were observed between mice that received microbiota from young donor mice and those that received microbiota from old donor mice (data not shown). However, analysis of relative abundances revealed that the amount of several bacterial genus differs between the two groups of mice in the *Bacteroidetes, Firmicutes* and *Proteobacteria* phyla. The proportions of *Ruminococcaceae, Acetatifactor, Lachnospira, Lachnospiraceae, Peptococcus*, and *Escherichia-Shigella* were significantly increased and those of *Eubacterium ventriosum, Eubacterium xynalophilum, Christensenellaceae, Rikenellaceae*, and *Candidatus arthromitus* were significantly decreased in the fecal microbiota of mice inoculated with the aged microbiota compared to those that received the young microbiota (Figure 1B). These results indicate that, 60 days after the inoculation, the composition of the microbiota in mice transplanted with the microbiota of old donors is distinct from that of mice that received a young microbiota.

### Gut Microbiota Has an Impact on the Cortex Content in Cholesterol and Phospholipids

**Figure 2:**
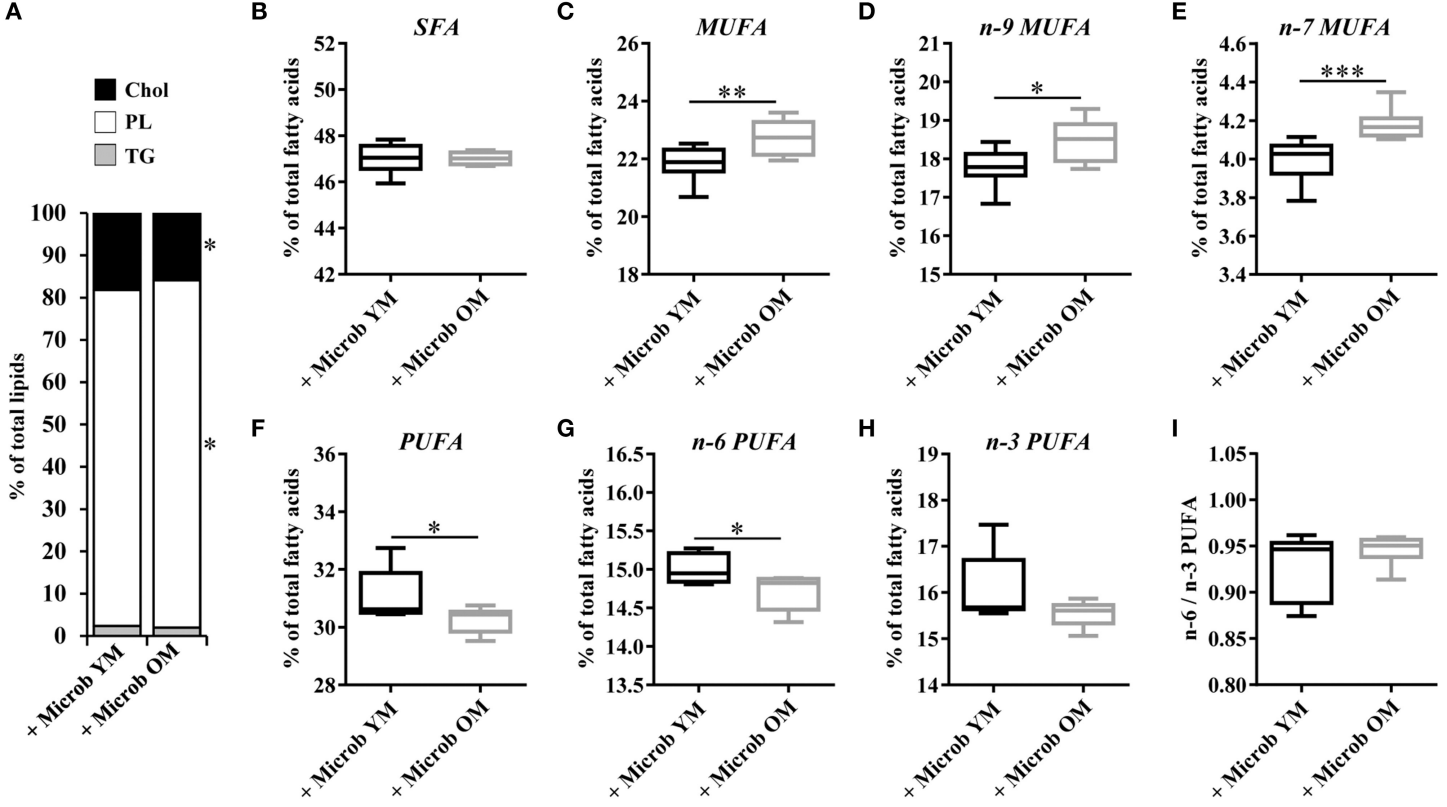
Relative abundance of lipid classes and ester-linked fatty acids in the cortex of mice harboring different age-related microbiota. Lipids were extracted from the cortex of mice inoculated with fecal microbiota of young mice (+ Microb YM) or old mice (+ Microb OM). **(A)** Relative abundance of lipid classes. Results are expressed as the percentages of cholesterol (Chol), phospholipids (PL), and triglycerides (TG) relative to total lipids, defined as 100%. **(B–H)** Relative abundance of ester-linked fatty acids. The results correspond to the quantification of fatty acid methyl esters derivatives (FAME) by GC-FID. They are expressed as the percentages of total **(B)** saturated fatty acids (SFA), **(C)** monounsaturated fatty acids (MUFA), **(D)** omega-9 (n-9) MUFA, **(E)** omega-7 (n-7) MUFA, **(F)** polyunsaturated fatty acids (PUFA), **(G)** omega-6 (n-6) PUFA and **(H)** omega-3 (n-3) PUFA relative to total fatty acids, defined as 100%. **(I)** Ratio of n-6/n-3 PUFA. **(B–I)** All data are presented as mean ± SEM. *n* = 7 for the group of mice inoculated with the microbiota of young mice and *n* = 8 for the group of mice inoculated with the microbiota of old mice. Comparisons were done between the two groups of mice (+ Microb YM and + Microb OM). *p < 0.05, **p < 0.01 and ***p < 0.001.

In order to investigate whether changes in the gut microbiota modulate the lipid content of the cortex, we performed several series of lipid analyses. Within tissue lipid classes, the amount of triglycerides (TG) was unchanged by the microbiota (Figure 2A). However, a significant increase in the amount of total phospholipids and a significant decrease in cholesterol were observed in mice transplanted with an aged microbiota compared to those inoculated with a young microbiota (2.7 ± 0.3% and 2.3 ± 0.3%, respectively) (Figure 2A). As cholesterol in the adult brain is primarily supplied by *de novo* synthesis, we analyzed the expression levels of the *Hmgcs1* gene, which encodes the hydroxymethylglutaryl-CoA synthase (HMG-CoA synthase), the enzyme catalyzing the reaction in which acetyl-CoA condenses with acetoacetyl-CoA to form HMG-CoA, and *Hmgcr* gene, which encodes the HMG-CoA reductase, the enzyme responsible for converting HMG-CoA to mevalonate, a rate-limiting step in cholesterol synthesis (Zhang and Liu, 2015). No significant change in the mRNA levels of *Hmgcr* or *Hmgcs1* was observed (**Figures 4A,B**). These data suggest that changes in the gut microbiota composition that are associated with aging modify cholesterol content of the cortex by a mechanism other than the one involving the HMGCR and HMGCS enzymes.

### Fatty Acid Composition of the Cortex Is Modulated by the Gut Microbiota

**Figure 3:**
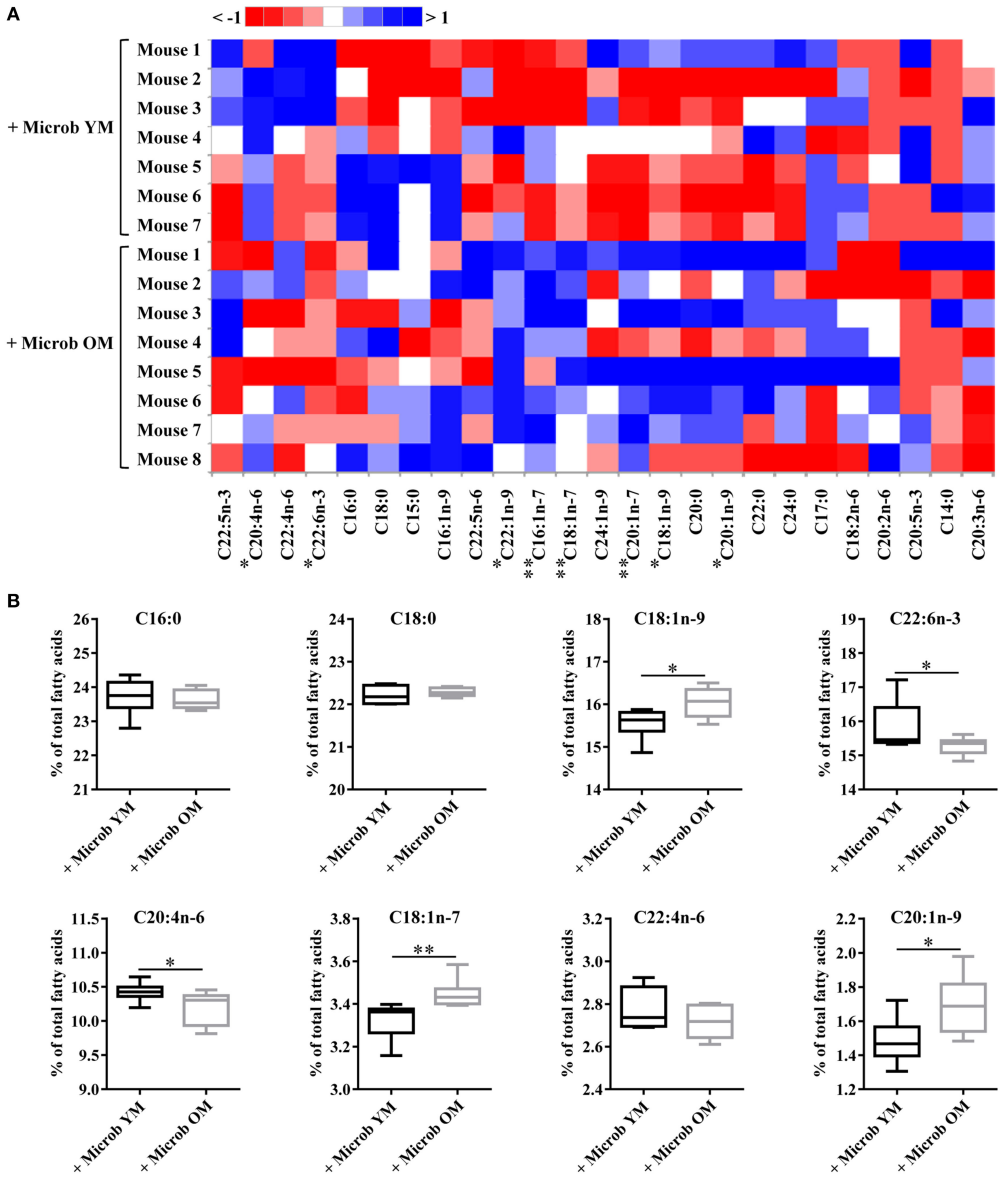
Ester-linked fatty acid profile in the cortex of mice inoculated with different age-related microbiota. Lipids were extracted from the cortex of mice inoculated with fecal microbiota of young mice (+ Microb YM) or old mice (+ Microb OM). The results correspond to quantification of fatty acid methyl esters (FAME) derivatives by GC-FID. The percentage of each fatty acid relative to that of total fatty acids (100%) was determined. **(A)** Heat map showing the abundance of each fatty acid relative to total fatty acids, defined as 100% **(B)** Relative abundance of the eight most abundant fatty acids quantified in the brain. All data are presented as mean ± SEM. *n* = 7 for the group of mice inoculated with the microbiota of young mice and *n* = 8 for the group of mice inoculated with the microbiota of old mice. Comparisons were done between the two groups of mice (+ Microb YM and + Microb OM). *p < 0.05 and **p < 0.01.

Effect of age-related microbiota on the fatty acid composition of the cortex was investigated by GC-FID analyses. The amount of each fatty acid was determined relative to total fatty acids. Regardless of the inoculated microbiota origin, no changes were observed in the abundance of saturated fatty acids (SFA) (Figures 2B, 3 and Supplementary Table 2). However, a significant increase in the amount of total monounsaturated fatty acids (MUFA) was measured in the cortex of mice inoculated with the aged microbiota compared to those of mice with the young microbiota (22.7 ± 0.2% vs. 21.8 ± 0.2% of total fatty acids, respectively) (Figure 2C). Interestingly, two series of MUFA, namely the omega-7 (n-7) and omega-9 (n-9) series, were significantly altered by the age-related changes in gut microbiota (Figures 2D,E, 3 and Supplementary Table 2). Among the 8 most quantitatively represented fatty acids that we detected in the mouse cortex, 3 of them were MUFA (C18:1n-9, C18:1n-7, and C20:1n-9) and their abundances were significantly increased in mice inoculated with the aged microbiota compared to those transplanted with the young one (Figure 3B).

Our data also revealed that microbiota modulates the amount of tissue polyunsaturated fatty acids (PUFA) (Supplementary Table 2). A significantly reduced amount of total PUFA was observed in mice inoculated with the microbiota of old mice compared to those transplanted with young microbiota (30.2 ± 0.2% and 31.2 ± 0.3%, respectively) (Figure 2F). Among them, both n-6 and n-3 series were impacted by the microbiota, but only total n-6 were significantly altered (Figures 2G,H). Particularly, the levels of the most represented PUFA in the brain, arachidonic acid (C20:4n-6 or AA) and docosahexaenoic acid (C22:6n-3 or DHA), were significantly reduced in mice transplanted with an aged microbiota (Figure 3B). The ratio of omega-6 PUFA to omega-3 PUFA remained unchanged (Figure 2I).

**Figure 4:**
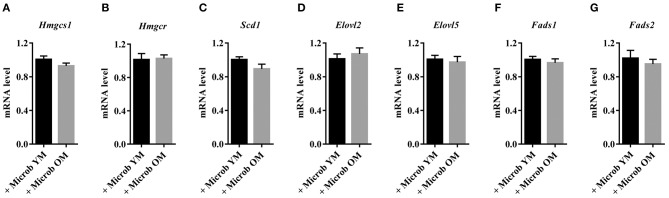
Cortical expression of enzymes involved in cholesterol synthesis in mice harboring age-dependent microbiota. **(A,B)** Cortical expression of genes encoding enzymes involved in the biosynthesis of cholesterol: **(A)** hydroxymethylglutaryl-CoA synthase (*Hmgcs1*) and **(B)** 3-hydroxy-3-methylglutaryl-CoA reductase (*Hmgcr*). **(C–G)** Cortical expression of genes encoding enzymes involved in the biosynthesis of fatty acids: **(C)** acyl-CoA desaturase 1 (*Scd1*), **(D,E)** elongation of very long chain fatty acids proteins 2 and 5 (*Elovl2* and *Elovl5*), **(F)** acyl-CoA (8-3)-desaturase (*Fads1*) and **(G)** acyl-CoA 6-desaturase (*Fads2*). The levels of mRNA were normalized to *Hprt* mRNA level for calculation of the relative levels of transcripts. mRNA levels are illustrated as fold change. All data are presented as mean ± SEM. *n* = 7 for the group of mice inoculated with the microbiota of young mice and *n* = 8 for the group of mice inoculated with the microbiota of old mice.

With the exception of the essential fatty acids, the linoleic acid (C18:2n-6) and alpha-linolenic acid (C18:3n-3) that are only provided through diet, brain fatty acids can be synthesized by *de-novo* lipogenesis in brain cells (Tracey et al., 2018). Therefore, we investigated whether the modifications in the content of cortex MUFA and PUFA were the result of dysregulations in the expression of enzymes involved in fatty acid biosynthesis in this tissue. We found that the expression levels of enzymes involved in MUFA biosynthesis (*Scd1*) and PUFA unsaturation (*Fads1* and *Fads2*) and elongation (*Elovl2* and *Elovl5*) were not impacted by the composition of the gut microbiota (Figure 4).

Altogether, these data show that age-related changes in gut microbiota promote profound modifications of the fatty acid profile in the cortex.

### Identification of Lipid Species Modified in the Cortex by Age-Associated Changes in the Gut Microbiota

Because cortex fatty acids are almost exclusively esterified on phospholipids, and since phospholipid subclasses play differential roles in brain metabolism (Farooqui et al., 2000; Tracey et al., 2018), we wanted to find out which phospholipids were involved in the changes we observed in the fatty acid composition of the cortex. HPLC-MS^2^ analyses revealed the presence of 41 species of phosphatidyl and plasmenylcholine (PC and PlsC), 80 species of phosphatidyl, and plasmenylethanolamine (PE and PlsE), 14 species of phosphatidyl and plasmenylinositol (PI and PlsI) and 15 species of sphingolipids, which were all sphingomyelins (SM) in the cortex (Supplementary Tables 3, 4). We compared, for each phospholipid class, the relative abundance of lipid species based on the fatty acid (SFA, MUFA, PUFA, C20:4, or C22:6) esterified at the sn-2 position (Table 1). Although the cortex content in total SFA was not modified by age-associated changes in the gut microbiota composition, we observed several alterations in the distribution of SFA among phospholipid classes and at the species level. On one hand, mice inoculated with the microbiota of old mice had a very slight but significant increase in the amount of PlsE with SFA at the sn-2 position compared to those inoculated with the microbiota of young mice (0.80 ± 0.01% and 0.76 ± 0.01%, respectively) (Table 1). These changes affected the following PlsE species: PlsE (18:1p/18:0), PlsE (16:0p/20:0), PlsE (18:1p/20:0) and PlsE (18:0p/20:0) (Supplementary Table 3). On the other hand, SM with SFA were significantly decreased by 1.2 ± 0.4% in the cortex of mice inoculated with an aged microbiota compared to those inoculated with a young microbiota (Table 1). These microbiota-dependent modifications affected the SM (d18:1/17:0) and SM (d18:1/18:0) species, the latter representing around 50% of the total SM species (Supplementary Table 4).

**Table 1:** Relative amounts of lipid species in the cortex depending on the lipid class and the family of fatty acids esterified at the sn-2 position.

	**+ Microb YM (*n =* 7)**	**+ Microb OM (*n =* 8)**
**Choline Glycerophospholipids (Non-hydrolyzed Form)**		
***Phosphatidylcholine (PC) and/or plasmenylcholine (PlsC)***		
PC (sn-2 SFA)	29.357 ± 0.184	29.039 ± 0.188
PC/PlsC (sn-2 MUFA)	49.363 ± 0.186	49.643 ± 0.151
PC/PlsC (sn-2 PUFA)	36.891 ± 0.114	37.062 ± 0.194
PC (sn-2 C20:4)	11.616 ± 0.059	11.324 ± 0.147
PC (sn-2 C22:6)	19.261 ± 0.141	19.576 ± 0.220
**Lysophosphatidylcholines (LPC)**		
LPC SFA	67.589 ± 0.703	69.306 ± 0.720
LPC MUFA	21.358 ± 0.350	20.688 ± 0.397
LPC PUFA	11.053 ± 0.396	10.006 ± 0.379
LPC C20:4	5.637 ± 0.261	4.778 ± 0.256
LPC C22:6	4.474 ± 0.159	4.312 ± 0.154
**Ethanolamine Glycerophospholipids (Non-hydrolyzed Form)**		
***Phosphatidylethanolamine (PE)***		
PE (sn-2 SFA)	0.914 ± 0.017	0.907 ± 0.017
PE (sn-2 MUFA)	11.447 ± 0.141	11.878 ± 0.178
PE (sn-2 PUFA)	58.945 ± 0.250	57.838 ± 0.318
PE (sn-2 C20:4)	18.002 ± 0.160	17.524 ± 0.262
PE (sn-2 C22:6)^*^	29.879 ± 0.203	29.073 ± 0.196
***Plasmenylethanolamine (PlsE)***		
PlsE (sn-2 SFA)^*^	0.755 ± 0.010	0.799 ± 0.012
PlsE (sn-2 MUFA)	7.093 ± 0.189	7.812 ± 0.231
PlsE (sn-2 PUFA)	19.543 ± 0.100	19.314 ± 0.160
PlsE (sn-2 C20:4)	4.559 ± 0.038	4.531 ± 0.056
PlsE (sn-2 C22:6)	9.473 ± 0.081	9.238 ± 0.092
**Lysophosphatidylethanolamines (LPE)**		
LPE SFA^*^	12.160 ± 0.262	13.137 ± 0.202
LPE MUFA	20.907 ± 0.638	22.261 ± 0.681
LPE PUFA^*^	66.933 ± 0.523	64.602 ± 0.678
LPE C20:4^*^	17.629 ± 0.160	16.888 ± 0.262
LPE C22:6^*^	48.937 ± 0.423	47.363 ± 0.465
**Inositol Glycerophospholipids (Non-hydrolyzed Form)**		
***Phosphatidylinositol (PI)***		
PI (sn-2 SFA)	1.162 ± 0.083	1.292 ± 0.036
PI (sn-2 MUFA)	4.956 ± 0.200	5.356 ± 0.146
PI (sn-2 PUFA)	82.667 ± 0.203	83.082 ± 0.311
PI (sn-2 C20:4)	79.187 ± 0.213	79.285 ± 0.243
PI (sn-2 C22:6)	3.170 ± 0.084	3.457 ± 0.157
***Plasmenylinositol (PlsI)***		
PlsI (sn-2 C20:4)	11.215 ± 0.226	10.270 ± 0.409
**Sphingomyelins (SM)**		
SM (sn-2 SFA)^*^	90.794 ± 0.242	89.571 ± 0.432
SM (sn-2 MUFA)	15.342 ± 0.362	16.636 ± 0.518

Lipids were extracted from the cortex of mice inoculated with gut microbiota of young mice (+ Microb YM) or old mice (+ Microb OM). Lipid species were analyzed by LC-MS^2^. Results are expressed as abundance (in percentage) of each species relative to that of total species in the lipid class to which it belongs (100%). It should be noted that for the choline phospholipids and for certain masses, some species could not clearly be identified. As a consequence, the distinction between PC and PlsC cannot be made and the percentages presented here include the different possible combinations. The data of the lipid species for which the fatty acids have not been attributed to the sn-1 and sn-2 positions have been removed from this analysis. Data are expressed as mean ± SEM.

* *p < 0.05*.

In addition to that, we observed that the amount of PE with C22:6 was significantly decreased by 0.8% ± 0.2% in the cortex of mice inoculated with the microbiota of old mice compared to those inoculated with the microbiota of young mice (Table 1). These changes were highlighted particularly by alterations in the PE (20:0/22:6) and PE (18:0/22:6) species, the latter representing about 20% of the total PE and PlsE species and 50% of the PE and PlsE species esterified to C22:6n-3 (Supplementary Table 3).

Among PC/PlsC and PI/PlsI classes, comparison of lipid species abundance based on the fatty acid (SFA, MUFA, PUFA, C20:4, or C22:6) esterified at the sn-2 position showed no significant difference between the two groups of mice (Table 1). However, at the level of lipid species we observed a slight but significant decrease of 0.4 ± 0.2% in the amount of PC (16:0/18:1). This species has a MUFA esterified at the sn-2 position and represents 22.5 ± 0.1% of the PC and PlsC in the cortex of mice inoculated with an aged microbiota (Supplementary Table 3). The amounts of 5 others PC species were significantly modified between the two groups of mice but, altogether, they represent <0.1% of the PC and PlsC (Supplementary Table 3).

Lysophospholipids are phospholipids in which one of the fatty acyl chains has been removed and replaced with a hydroxyl group. They can reflect a degradation of phospholipids in tissues. Eight species of lysophosphatidylcholines (LPC) and 5 species of lysophosphatidylethanolamines (LPE) were detected (Supplementary Table 3). Comparison of the abundance of lysophospholipid individual species revealed several changes but only among LPE series (Table 1). A significant increase in the amount of LPE esterified to SFA and a significant decrease in LPE with PUFA (including LPE C20:4 and LPE C22:6) were observed in the brain of mice harboring the microbiota of old mice compared to those with the microbiota of young mice (Table 1 and Supplementary Table 3).

These data show that changes in the gut microbiota composition associated with aging has an impact on the relative abundance of cortex individual phospholipid species, and particularly on PC/PlsC, PE/PlsE, LPE, and SM.

### Plasma Lipid Composition Is Altered in Mice Inoculated With the Microbiota of Old Mice

**Figure 5:**
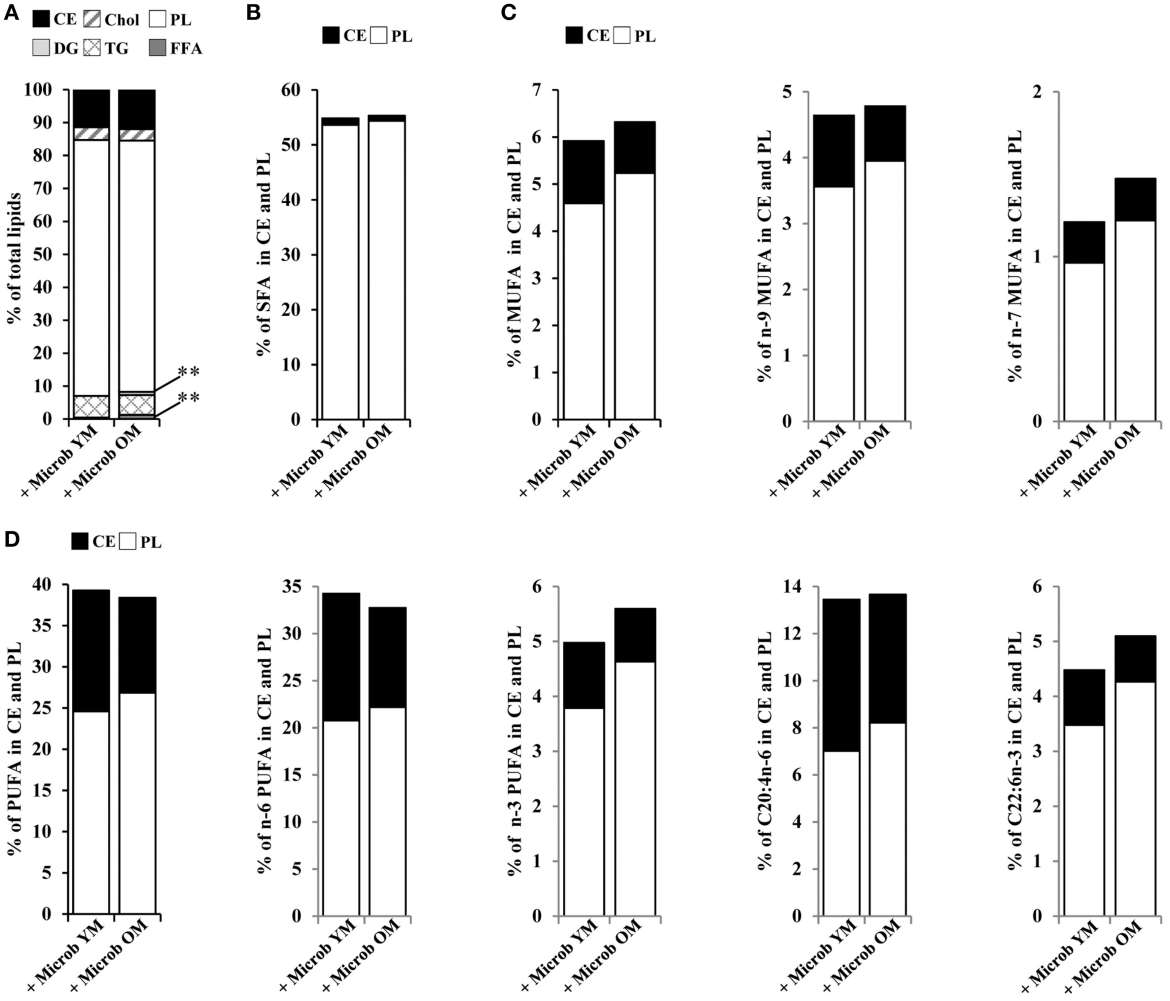
Plasma lipid and fatty acid profile in mice inoculated with different age-related microbiota. Lipids were extracted from the plasma of mice inoculated with fecal microbiota of young mice (+ Microb YM) or old mice (+ Microb OM). **(A)** Relative abundance of lipid classes. Results are expressed as the percentages of cholesteryl esters (CE), cholesterol (Chol), phospholipids (PL), diglycerides (DG), triglycerides (TG), and free fatty acids (FFA) relative to total lipids, defined as 100%. **(B–D)** Total lipid extract was separated on silica gel. CE and PL were extracted and their fatty acid profiles were determined by GC-FID. To express the results, we took into account the relative proportions of CE and PL measured in the total lipid extracts. Results are expressed as percentages of **(B)** total saturated fatty acids (SFA), **(C)** monounsaturated fatty acids (MUFA), and **(D)** polyunsaturated fatty acids (PUFA) on CE and PL in total lipid extracts, defined as 100%. All data are presented as mean. *n* = 7 for the group of mice inoculated with the microbiota of young mice and *n* = 7 for the group of mice inoculated with the microbiota of old mice. **p < 0.01.

Fatty acids are taken up into the brain from circulating blood by mechanisms that still remain under debate (Katz et al., 2007; Bruce et al., 2017). Our data showed that the relative amounts of plasma cholesteryl ester, cholesterol, triglycerides, and phospholipids were not modified by changes in the gut microbiota (Figure 5A). However, mice inoculated with microbiota of old mice exhibited significantly increased amounts of plasma diglycerides (0.9 ± 0.3% vs. 0.0 ± 0.0%) and free fatty acids (1.3 ± 0.2% vs. 0.5% ± 0.2%) compared to mice inoculated with the microbiota of young mice, suggesting a partial hydrolysis of blood triglycerides (Figure 5A). These results suggest that the plasma lipid composition is modified by an aged microbiota.

By GC-FID, we evaluated more specifically the variations in the distribution of fatty acids on cholesteryl esters and phospholipids (Figures 5B–D). No significant difference in the amounts of fatty acid series or DHA and AA was observed between the groups of mice that have received different age-related microbiota. However, not being significant and limited to cholesteryl esters and phospholipids, these data do not allow us to conclude on an effect due to changes in the composition of the intestinal microbiota on the fatty acid content of the plasma.

### Age-Related Changes of Gut Microbiota Modulate Hepatic Lipogenesis

**Figure 6:**
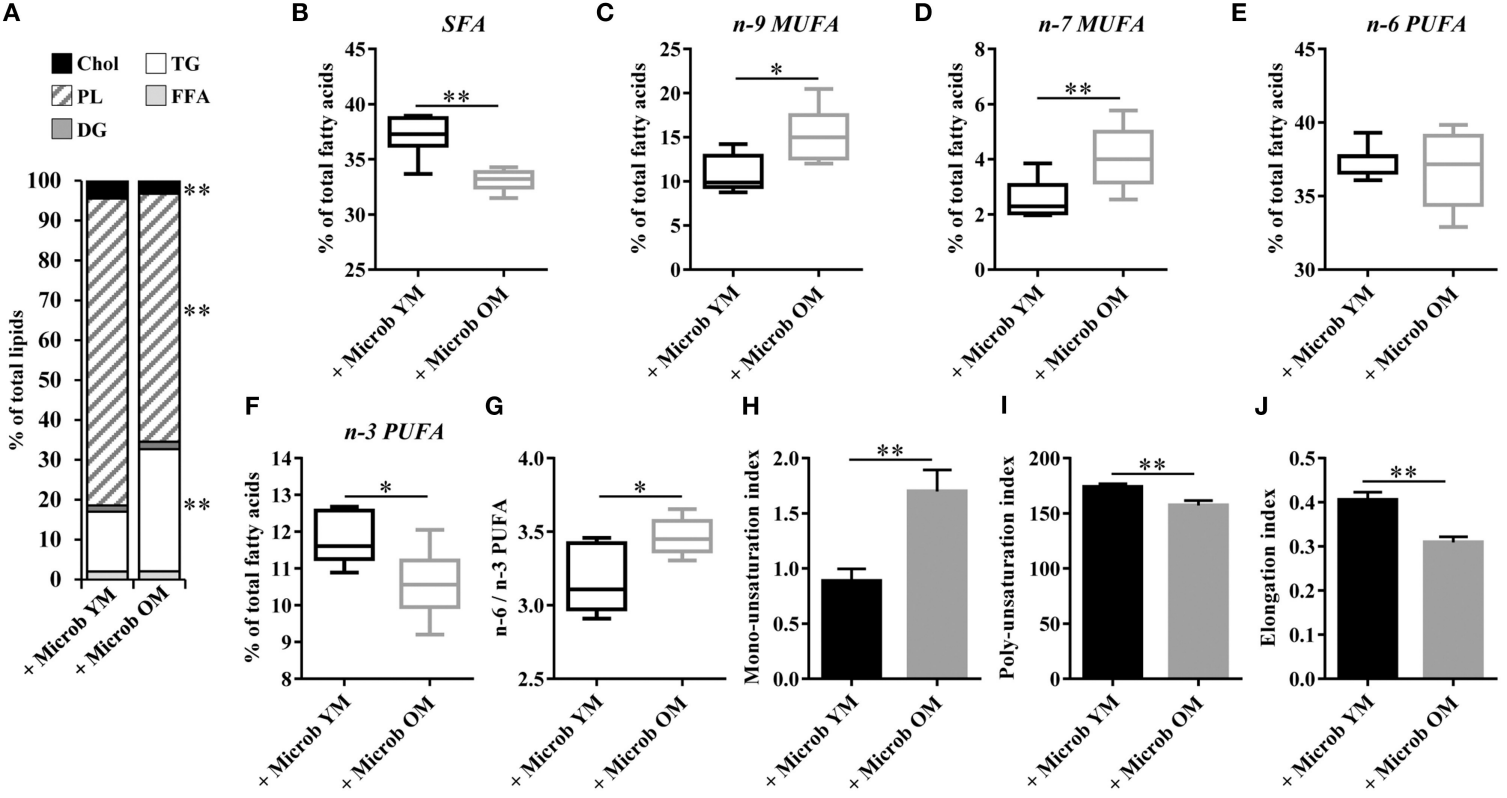
Relative abundance of lipid classes and ester-linked fatty acids in the liver of mice inoculated with different age-related microbiota. Lipids were extracted from the liver of mice inoculated with fecal microbiota of young mice (+ Microb YM) or old mice (+ Microb OM). **(A)** Relative abundance of lipid classes. Results are expressed as the percentages of cholesterol (Chol), phospholipids (PL), diglycerides (DG), triglycerides (TG), and free fatty acids (FFA) relative to total lipids, defined as 100%. **(B–J)** The results presented were obtained from the analysis of the quantification data of fatty acid methyl esters (FAME) derivatives by GC-FID. Results are expressed as the percentages of total **(B)** saturated fatty acids (SFA), **(C)** omega-9 (n-9) monounsaturated fatty acids (MUFA), **(D)** omega-7 (n-7) MUFA, **(E)** omega-6 (n-6) polyunsaturated fatty acids (PUFA), and **(F)** omega-3 (n-3) PUFA relative to total fatty acids, defined as 100%. **(G)** Ratio of n-6/n-3 PUFA. **(H)** Mono-unsaturation index corresponding to the sum of the ratios “products” (C16:1n-7 and C16:1n-9)/“precursor” (C16) and “products” (C18:1n-7 and C18:1n-9)/“precursor” (C18). **(I)** Poly-unsaturation index corresponding to the sum of the amounts of each fatty acid that had more than on carbon-carbon double bond multiplied by its unsaturated bond number. **(J)** Elongation index corresponding to the ratio of fatty acids with 20-carbons and more/fatty acids with 16- and 18-carbons. All data are presented as mean ± SEM. *n* = 7 for the group of mice inoculated with the microbiota of young mice and *n* = 8 for the group of mice inoculated with the microbiota of old mice. Comparisons were done between the two groups of mice (+ Microb YM and + Microb OM). *p < 0.05 and **p < 0.01.

We investigated whether alterations in the cortex lipid composition occurring in mice that received the microbiota of old donor mice could be the consequence of an altered hepatic lipid metabolism. Our data revealed profound modifications of the liver lipid composition in mice inoculated with the microbiota of old mice comparatively to those inoculated with the microbiota of young mice (Figure 6A). These changes were characterized by significant decreases in the amounts of cholesterol (3.2 ± 0.2% vs. 4.5 ± 0.3%) and phospholipids (62.2 ± 2.4% vs. 77.0 ± 2.9%), balanced by a significant increase in the amount of triglycerides (30.7 ± 2.7% vs. 15.0 ± 3.0%). The analysis of the hepatic fatty acid composition by GC-FID also showed remarkable microbiota-dependent changes (Figure 6). The amount of SFA was significantly reduced (by 4.0 ± 0.3%) and that of MUFA was significantly increased (by 6.0 ± 1.5%) in mice with a microbiota of old mice compared to those with a young microbiota (Figures 6B–D). Regarding PUFA, the amount of n-6 fatty acids was unchanged whereas that of the n-3 fatty acids was significantly decreased in mice with an aged microbiota (10.6 ± 0.3% vs. 11.8 ± 0.3%), resulting in a significant increase in the n-6 PUFA to n-3 PUFA ratio (Figures 6E–G).

**Figure 7:**
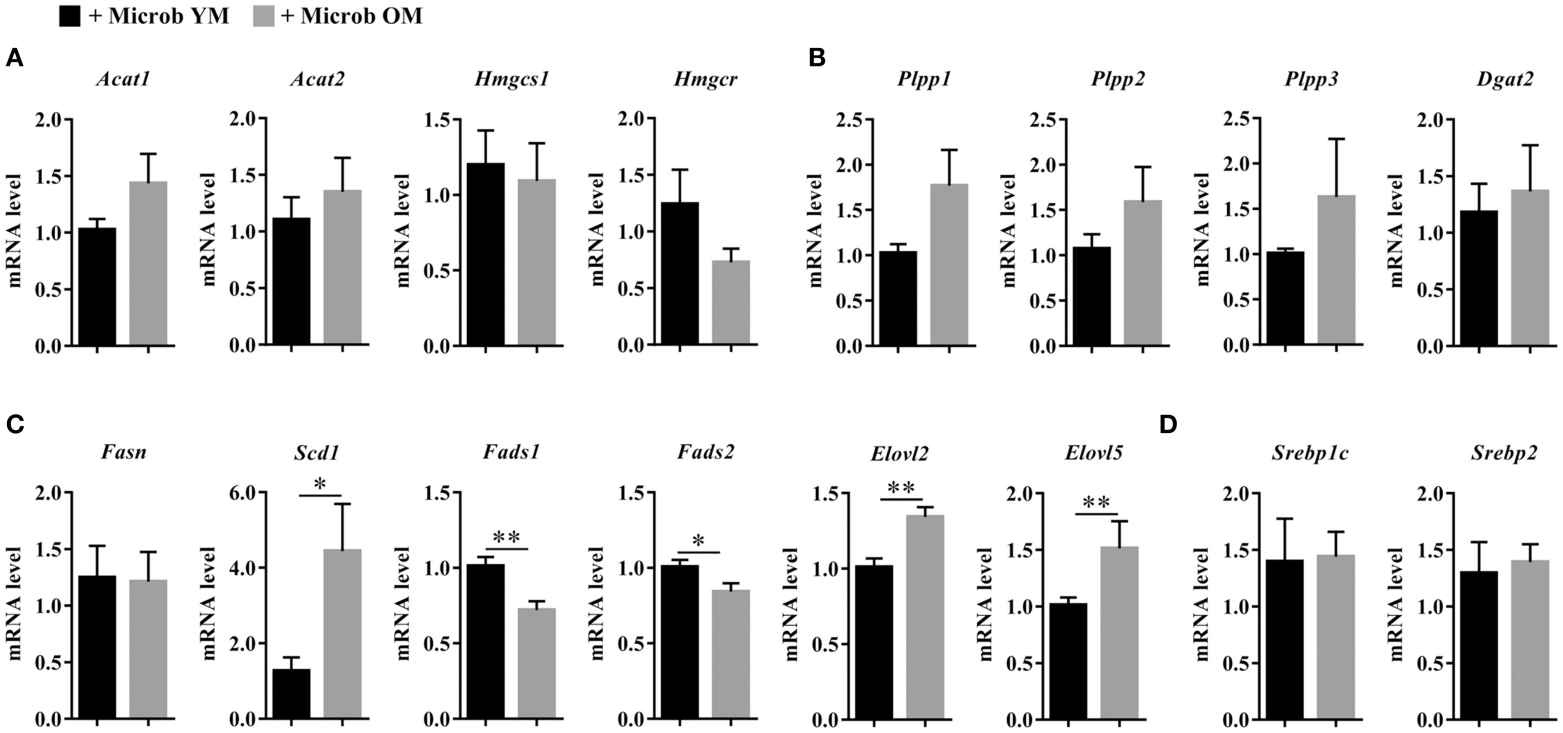
Expression of hepatic genes involved in lipid biosynthesis. **(A)** Hepatic expression of genes encoding enzymes involved in the biosynthesis of cholesterol: acetyl-CoA acetyltransferase 1 and 2 (*Acat1* and *Acat2*), hydroxymethylglutaryl-CoA synthase (*Hmgcs1*) and 3-hydroxy-3-methylglutaryl-CoA reductase (*Hmgcr*). **(B)** Hepatic expression of genes encoding enzymes involved in the biosynthesis of di- and triglycerides: phospholipid phosphatases 1, 2, and 3 (*Plpp1, Plpp2* and *Plpp3*) and diacylglycerol O-acyltransferase 2 (*Dgat2*). **(C)** Hepatic expression of genes encoding enzymes involved in the biosynthesis of fatty acids: fatty acid synthase (*Fasn*), acyl-CoA desaturase 1 (*Scd1*), acyl-CoA (8-3)-desaturase (*Fads1*), acyl-CoA 6-desaturase (*Fads2*), and elongation of very long chain fatty acids proteins 2 and 5 (*Elovl2* and *Elovl5*). **(D)** Hepatic expression of genes encoding enzymes involved in the regulation of lipid synthesis: sterol regulatory element-binding proteins 1 (variant 3) and 2 (*Srebp1c* and *Srebp2*). The levels of mRNA were normalized to *Hprt* mRNA level for calculation of the relative levels of transcripts. mRNA levels are illustrated as fold change. All data are presented as mean ± SEM. *n* = 7 for the group of mice inoculated with the microbiota of young mice and *n* = 8 for the group of mice inoculated with the microbiota of old mice. Comparisons were done between the two groups of mice (+ Microb YM and + Microb OM). *p < 0.05 and **p < 0.01.

We next investigated whether changes in the hepatic lipid content observed in mice inoculated with the microbiota of old donors could be due to an altered expression of genes involved in lipogenesis. Hepatic expression of genes encoding enzymes involved in the biosynthesis of cholesterol (*Acat1, Acat2, Hmgcs1*, and *Hmgcr*), di- and triglycerides (*Plpp1, Plpp2, Plpp3*, and *Dgat2*) and fatty acids (*Fasn, Scd1, Fads1, Fads2, Elovl2*, and *Elovl5*), and in the regulation of lipid metabolism (*Srebp1c* and *Srebp2*) was measured (Figure 7). No significant modification of the transcript levels of genes involved in cholesterol, di- and triglycerides biosynthesis and in the regulation of hepatic lipid metabolism was observed (Figures 7A,B,D). However, we observed significant decreases in the expression of *Fads1* and *Fads2* (by 28.8 ± 5.8% and 16.3 ± 5.5%, respectively) in mice that have received the microbiota of old mice compared to those inoculated with the microbiota of young mice (Figure 7C). This result was in accordance with the significant decrease in the poly-unsaturation index observed in mice inoculated with an aged microbiota (Figure 6I).

In addition, the level of *Scd1* mRNA was significantly increased by 3.5 in mice harboring an aged microbiota compared to those with a young microbiota (Figure 7C). SCD1 (stearoyl-CoA desaturase 1) is the rate limiting enzyme that catalyzes the biosynthesis of MUFA. In accordance with *Scd1* mRNA level, we observed an increase in the mono-unsaturation index in mice inoculated with the microbiota of old mice compared to those inoculated with the microbiota of young mice (Figure 6H). The level of *Elovl2* and *Elovl5* mRNA were also significantly increased by 1.3 and 1.5, respectively, in mice harboring an aged microbiota compared to those with a young microbiota (Figure 7C). ELOVL2 (elongation of very long chain fatty acids protein 2) and ELOVL5 catalyze the reactions of the long-chain fatty acids elongation cycle. However, these results were unexpected since we observed a significant decrease in the hepatic elongation index in mice inoculated with the microbiota of old mice suggesting a decreased amount of long-chain fatty acids (Figure 6J).

Taken together, these results show that age-associated changes in microbiota promote a reprograming of genes related to MUFA biosynthesis and PUFA elongation and desaturation in the liver.

## Discussion

In humans, modifications in the gut microbiota during aging have been reported and a large number of studies suggest that these changes could be a major factor that can tip the balance between healthy and non-healthy aging. Since aging is associated with dysregulations in lipid metabolism, we investigated whether changes in the gut microbiota that are associated with aging can alter host lipid homeostasis.

Aging is a complex process affecting host physiological, genomic, metabolic, and immunological functions (Lopez-Otin et al., 2013). The dialogue between host and microbiota is bi-directional and alterations of the host can also shape the gut microbiota (Goodrich et al., 2014; Davenport, 2016; Kolde et al., 2018). In addition, in humans a large number of environmental factors such as the place of residence (hospital, residential care setting or home) or the diet can influence the composition of the gut microbiota. For that matter, multiple factors should be taken into account when studying the relationship of the gut microbiota with the host (O'Toole and Jeffery, 2015; Garcia-Pena et al., 2017). The germ-free mouse model offers a good opportunity to study the impact of gut microbes on host physiology by overcoming these variables. In order to study the impact of age-related changes of microbiota on lipid homeostasis, we transferred the fecal microbiota of young vs. old donor mice into germ-free mice. The composition of the fecal microbiota recovered in mice after 2 months of colonization was analyzed by 16S rRNA gene sequencing, a powerful tool widely used to explore the composition of bacterial communities (Rapin et al., 2017). We showed that microbiota were different between the two groups of mice, with that of mice inoculated with fecal microbiota of old donors recapitulating some features described in the elderly. This includes an increase in the relative abundance of *Escherichia-Shigella*, a bacterial genus associated with inflammation, and a decrease in *Christensenellaceae*, a bacterial family associated with longevity (Biagi et al., 2016; Kumar et al., 2016). However, it should be noticed that the 16S rRNA sequencing method has some limitations and gives little information about the whole genetic, functional, and metabolic capabilities of the microbiota (Rapin et al., 2017).

The brain tissue is characterized by its high content in lipids. In fact, lipids constitute approximately half of the brain dry weight and mainly consist of phospholipids, cholesterol and sphingolipids. Because of their high diversity and due to their hydrophobic properties, lipids play crucial roles in maintaining the structure and the functions of the brain. Alteration in the brain lipid metabolism is associated with many neurological disorders (Adibhatla and Hatcher, 2007). Cholesterol is essential for neuronal physiology (Zhang and Liu, 2015). During aging, its amount decreases in the cortex, and defects in cholesterol metabolism were reported to be associated with neurodegenerative diseases and cognitive deficits related to aging (Soderberg et al., 1990; Svennerholm et al., 1994; Zhang and Liu, 2015). Interestingly, our results reproduced this cholesterol alteration in the cortex of mice with the microbiota of old mice in comparison with the mice inoculated with the microbiota of young donor mice. As we have also observed a decrease in hepatic cholesterol amount, one can hypothesize that the decrease in brain cholesterol could be a consequence of decreased cholesterol availability in the bloodstream. But, such a hypothesis does not seem very plausible since, due to the blood brain barrier brain cholesterol is primarily produced through *de novo* synthesis (Dietschy and Turley, 2004). Another hypothesis could have been an alteration in the cholesterol biosynthesis pathway. However, we did not observe any modification in the expression of the rate-limiting enzyme in cholesterol synthesis HMGCR in the cortex of mice inoculated with the microbiota of old mice compared to those inoculated with the microbiota of young mice. To explain the decrease in cholesterol in mice with an aged microbiota we finally hypothesize that expression of cholesterol-removing enzymes could be enhanced (Martin et al., 2010; Nes, 2011). This hypothesis is supported by studies showing increased amount of cholesterol-24-hydroxylase enzyme (CYP46A1)—the major enzyme involved in cholesterol removal in neuronal tissues—in brains of old humans and rodents compared to young ones (Lund et al., 1999; Martin et al., 2008; Perovic et al., 2009).

Brain contains high amounts of phospholipids (glycerophospholipids and sphingomyelins) (O'Brien and Sampson, 1965; Soderberg et al., 1990). Glycerophospholipids are enriched in PUFA, particularly in docosahexaenoic acid (DHA, C22:6n-3) and arachidonic acid (AA, C20:4n-6) whereas sphingomyelins are composed of fatty acids with low levels of unsaturation. These lipids are structurally and functionally important cell constituents. Besides providing structural integrity to neuronal cell membranes for the proper functioning of membrane-bound enzymes or transport systems, they play many others important functions such as being reservoirs of signaling messengers and antioxidant molecules (Farooqui et al., 2000; Tracey et al., 2018). Any changes in the phospholipid content, whether in the distribution of different sub-classes or in their structure, could then have profound consequences on cell function. In this study, we showed that intestinal colonization by a microbiota from old donor mice compared to that of young donor mice profoundly modifies the structure of brain phospholipids by changing their fatty acid composition. Among the changes observed, there was an increased amount of MUFA belonging to the n-7 and n-9 series, mainly C18:1n-9, C18:1n-7, and C20:1n-9, which are part of the 8 most represented fatty acids in the brain. These changes were balanced by a decreased brain content in PUFA, and particularly in C20:4n-6 (arachidonic acid, AA) and C22:6n-3 (docosahexaenoic acid, DHA), the two major PUFAs in neurons. Interestingly, similar age-associated changes in fatty acid composition have already been reported in rat and human cortical tissues (Bowen et al., 1973; Favrelere et al., 2000; Giusto et al., 2002; Little et al., 2007; McNamara et al., 2008).

Ethanolamine and choline glycerophospholipids (PE and PC) are the two most represented classes of phospholipids in the brain, notably in cortical tissue (Sastry, 1985; Little et al., 2007). Our lipidomic data suggest that an aged microbiota is associated with changes in MUFA content in PE and PUFA content in PE and LPE. Increased amounts of PE esterified with C18:1n-9 and C20:1n-9 and/or decreased amount of PE esterified with C20:4n-6 or C22:6n-3 have previously been reported in the cortex of aged rats and macaques (Favrelere et al., 2000; Little et al., 2007; Latour et al., 2013; Lam et al., 2016). However, other authors did not observe any age-related modification of these PE species in old rat cortex (Letondor et al., 2014). Interestingly, and as already reported by Little et al. we show in our study a decrease in PE (18:0/22:6), which is the most represented species (about 20%) among all ethanolamine phospholipids (Little et al., 2007). Modifications among sphingomyelin species have also occurred in the cortex of mice harboring the microbiota of old donors compared to those that have received the microbiota of young donors. They affected two of the three major species of SM. The amount of SM (d18:1/18:0) that represents about half of the total SM species was decreased and this was balanced by SM (d18:1/24:1) that represents about 8% of the SM species and whose amount was increased in mice with an aged microbiota compared to those with a young one. Increase in SM (C24:1) has already been described in the cortex of old macaques and rats (Giusto et al., 1992; Lam et al., 2016).

The mechanisms of fatty acid uptake by the brain are not yet fully understood (Qi et al., 2002; Katz et al., 2007). Some specificities have been documented depending on the fatty acid and this explains, in part, the unique lipid profile of the brain, characterized by an enrichment in PUFA, and particularly in DHA and AA (Gazzah et al., 1994; Contreras et al., 2000; Rosenberger et al., 2010; Chen et al., 2013). Uptake and transport of fatty acids will also depend on their form (non-esterified or esterified), and on the lipid entities on which they are esterified. Identifying the fatty acids esterified on cholesterol and phospholipids in plasma did not allow us to clearly establish a link between circulating fatty acids and the brain fatty acid profile in mice harboring age-dependent microbiota. In this study, we have observed non-significant variations in the fatty acid composition of plasma CE and PL. Plasma total CE and PL originate from low-density lipoproteins (LDL) and high-density lipoproteins (HDL) making that the analysis of fatty acid composition of whole plasma CEs and whole plasma PLs considers LDL and HDL lipoproteins together. The variability and the non-significant tendencies we observed might then be the result of variations of the fatty acid composition in only one of these lipoprotein classes. One can hypothesize that targeting lipids of isolated LDL and/or HDL lipoproteins is likely to underline differences in the fatty acid composition between animals receiving an aged microbiota vs. a young microbiota, and then reinforce the hypothesis of defective liver biosynthesis and consequences on nervous tissues through blood transport. Further studies are needed to verify this hypothesis. In addition, we have observed an increase in the amount of FFA and DG in plasma of mice colonized by an aged microbiota. Increase of plasma FFA level have already been reported in metabolic and immune-related diseases and some data support a link between microbial dysbiosis and altered serum FFA levels (Boden, 2008; Rodriguez-Carrio et al., 2017). Beyond the influence that they could have on the fatty acid composition of the brain, attention should be paid to plasma FFA since they are likely to be degradation products of TG and could constitute systemic mediators modulating inflammation and metabolism, two conditions related to aging (Pararasa et al., 2015; Suiter et al., 2018).

Our data show that changes in microbiota influence the relative proportion of lipid classes in the liver. Indeed, mice that received the microbiota of old donors displayed elevated amount of TG that was balanced by reduced amount of phospholipids and cholesterol compared to mice inoculated with the microbiota of young mice. We further analyzed the expression of several genes encoding enzymes involved in the biogenesis of TG and cholesterol, but we did not observe any alteration. A hypothesize to explain the decrease in hepatic cholesterol in mice inoculated with the microbiota of old donors could be an enhanced conversion of cholesterol to bile acids. Indeed, bile acids are synthesized from cholesterol by hepatocytes and gut microbiota is involved in the regulation of bile acid synthesis, in particular by modulating the hepatic expression of the cholesterol 7α-hydroxylase (CYP7A1) (Sayin et al., 2013).

Moreover, the fatty acid profile observed in the liver was almost entirely similar to that of the brain. Except for SFA, whose amount was unchanged in the brain and decreased in the liver, transfer of an aged microbiota to mice induced an increase in the level of MUFA and a decrease in the level of n-3 PUFA compared to mice harboring a young microbiota. Accordingly, the mono-unsaturation index was augmented and the poly-unsaturation index was reduced in mice with an aged microbiota. Several studies support the role of gut microbes as potent regulators of hepatic lipid metabolism. Indeed, comparison of hepatic fatty acid metabolism between germ-free and conventionally raised specific-pathogen-free mice has shown that gut microbes impact the MUFA content and PUFA elongation by modifying *Scd1* and *Elovl5* expression, respectively (Kindt et al., 2018). In addition, alteration of the expression level of genes involved in hepatic lipid metabolism (*Srebp1c, Acc, Fas, Scd1, Acat2, Dgat1, Dgat2, Hmgcs*, and *Hmgcr*) has been reported in TLR5-deficient mice (Singh et al., 2015). In our study, we showed that modifications of the hepatic content in MUFA and PUFA were associated with the dysregulated expression of genes involved in fatty acid biosynthesis (upregulation of *Scd1*, and downregulation of *Fads1*, and *Fads2* gene expression). Unexpectedly, we observed an increase in the expression level of *Elovl2 and Elovl5* genes that encode elongases in the liver of mice inoculated with the microbiota of old mice while the elongation index was decreased. Although these results seem inconsistent, similar data has already been described by others. Indeed, Wang et al. observed an increase in MUFA abundance associated with a decrease in PUFA abundance in the liver of obese mice while the expression of *Elovl5, Elovl6*, and *Scd1* were upregulated (Wang et al., 2006). The facilitation of hepatic elongation and desaturation processes involving ELOVL5 and SCD1 could contribute to and explain the increased production of hepatic MUFA.

Our study was conducted in male mice. However, studies in human and animal models have shown that sex dimorphism in lipid metabolism exists and that the phospholipid and fatty acid compositions of several tissues, including those of plasma, brain and liver, differ according to the gender (Childs et al., 2010; Slater-Jefferies et al., 2010; Varlamov et al., 2014; Rodriguez-Navas et al., 2016). Thus, it would be interesting to evaluate in another study whether an aged microbiota would induce in recipient female germ free mice similar changes in the fatty acid and phospholipid contents than those observed in males.

Metabolites generated by an aged microbiota and that promote the remodeling of lipid and fatty acid contents in the brain and in the liver remain to be identified. SCFA may constitute good candidates as they have been described to influence hepatic lipogenesis in addition to the part they take in the communication pathways between the gut and the brain (De Vadder et al., 2014; Singh et al., 2015; Dinan and Cryan, 2017; Kindt et al., 2018). In our study, alteration in the cortex lipid content due to gut colonization by an aged microbiota seems to reflect those observed in the liver, the hub of lipid metabolism. Identifying and acting on the microbial mediators involved in the dysregulation of hepatic lipogenesis might help develop strategies to influence brain lipid composition and prevent age-related lipid modifications of this tissue.

In conclusion, this study provides evidence that gut microbiota should also be considered as an important contributor to lipid changes occurring in the brain during aging. Changes in the gut microbiota related to aging is associated with modifications in lipid classes (in particular in reducing cholesterol amounts) and in fatty acid profile (in decreasing PUFA and among them DHA, and in increasing MUFA content) in the cortex. Such alterations have been described as features of cerebral aging. Given the crucial role played by these lipids in neuronal membrane dynamics but also in cell signalization, these microbiota-driven alterations of lipids could have profound impact on brain physiology.

Alterations of the hepatic lipid profile and fatty acid synthesis were also shown in response to gut colonization by an aged microbiota. This hepatic phenotype could be one of the drivers of the modification occurring in the brain but may also impact the metabolic activity of others organs. This point needs further studies.

Altogether, our results support the idea that manipulation of the microbiota holds promise as an innovative strategy to influence the development of comorbidities associated with aging.

## Data Availability Statement

All datasets generated for this study are included in the article/Supplementary Material.

## Ethics Statement

Experiments on animals were performed at the Anaxem platform (Micalis Institute, INRA, Jouy-en-Josas, France). The Anaxem facilities are accredited by the French “Direction Départementale de la Protection des Populations (DDPP78),” accreditation number A78-322-6. All procedures involving animal experimentation were carried out according to the European guidelines for the care and use of laboratory animals and were approved by the “French Ministère de l'Enseignement Supérieur et de la Recherche” (authorization number 3441-2016010614307552).

## Author Contributions

M-AB, PL, and NA designed the experiments. FC performed inoculation of germ-free mice with fecal microbiota. FC and MA collected the samples. CC and PL analyzed the data from microbiota sequencing. MA and BB performed gene expression analyses. SG and LM performed lipid class profiles and fatty acid analysis by gas chromatography. J-PP, SC, and OB performed brain lipid quantification by HPLC-MS^2^. MA, AB, NA, and M-AB analyzed the data and prepared the figures and tables. MA and M-AB wrote the manuscript. All authors reviewed the final version of the manuscript.

### Conflict of Interest

AB is consultant for Aerie, Allergan, Bausch & Lomb, Santen, and Théa Pharma. The remaining authors declare that the research was conducted in the absence of any commercial or financial relationships that could be construed as a potential conflict of interest.
