# Supplementary material for: Age-Related Changes in the Gut Microbiota Modify Brain Lipid Composition
Source: Front Cell Infect Microbiol. 2020 Jan 14;9:444. doi: 10.3389/fcimb.2019.00444 (PMC6970973; doi:10.3389/fcimb.2019.00444)
Supplement: Supplementary file 1 [file Data_Sheet_1.pdf]

Supplementary Table 1: Primers

Genes	Forward primers (5’-3’)	Reverse primers (5’-3’)
Acat1	CATCCAAGGGGGTGAAGGAC	CTGCCACCATCACATCCTGA
Acat2	CAAGGAGATTGTGCCAGTGC	CGTTCATTCCTGATGCGTTCG
Hmgcs1	CTGCAGTCTTCAATGCCGTG	AGGTCTGGCATTTCCTGTGG
Hmgcr	CATGACATTCTTCCCGGCCT	ATCCAGCGACTATGAGCGTG
Plpp1	CGGCAATCCCTACATAGCCA	AGATAGCCAAGAAGTGCGGC
Plpp2	TGAGGCCCACTGTTCAGTTC	AAACATAGCGGACCGTGAGG
Plpp3	ATTTCATGTGAGGGCCAGGG	TCTCTGATCCCAAGGAGCCA
Dgat2	CCAAGAAAGGTGGCAGGAGAT	TGGTCAGCAGGTTGTGTGTC
Fasn	GACTCGGCTACTGACACGAC	CGAGTTGAGCTGGGTTAGGG
Scd1	CAGGAGGGCAGGTTTCCAAG	CGTTCATTTCCGGAGGGAGG
Fads1	CGCCAAACGCGCTACTTTAC	CCACAAAAGGATCCGTGGCA
Fads2	CGTGGGCAAGTTCTTGAAGC	TCTGAGAGCTTTTGCCACGG
Elovl2	GTGATGTCCGGGTAGCCAAG	GGACGCGTGGTGATAGACAT
Elovl5	TGATGAACTGGGTTCCCTGC	CAGCTGCCCTTGAGTGATGT
Srebp1c	GGAGCCATGGATTGCACATT	GCTTCCAGAGAGGAGGCCAG
Srebp2	GCGATGGATGAGAGCAGCG	GTTGCCACTGCCACCATTG
Hprt	CAGTCCCAGCGTCGTGATTA	TGGCCTCCCATCTCCTTCAT

Supplementary Table 2: Relative amounts of ester-linked fatty acids in the cortex.

Fatty acids	+ Microb YM (n=7)	+ Microb OM (n=8)
**Saturated fatty acids**		
C14:0	0.090±0.002	0.091±0.002
C15:0	0.064±0.003	0.066±0.002
C16:0	23.741±0.199	23.633±0.098
C17:0	0.184±0.002	0.184±0.003
C18:0	22.231±0.078	22.283±0.034
C20:0	0.241±0.006	0.265±0.008
C22:0	0.233±0.013	0.251±0.010
C24:0	0.230±0.009	0.247±0.012
**Monounsaturated fatty acids**		
C16:1n-7**	0.443±0.005	0.468±0.004
C18:1n-7**	3.322±0.032	3.448±0.023
C20:1n-7**	0.231±0.008	0.266±0.006
C16:1n-9	0.146±0.003	0.148±0.003
C18:1n-9*	15.538±0.129	16.037±0.121
C20:1n-9*	1.488±0.050	1.695±0.059
C22:1n-9*	0.206±0.008	0.228±0.002
C24:1n-9	0.359±0.027	0.366±0.015
**Polyunsaturated fatty acids**		
C20:5n-3	0.085± 0.005	0.081±0.004
C22:5n-3	0.171±0.008	0.179±0.010
C22:6n-3*	15.941±0.279	15.287±0.089
C18:2n-6	0.833±0.016	0.832±0.031

C20:2n-6	0.167±0.002	0.182±0.009
C20:3n-6	0.552±0.004	0.536±0.011
C20:4n-6*	10.431±0.052	10.190±0.086
C22:4n-6	2.780±0.037	2.718±0.026
C22:5n-6	0.230±0.004	0.242±0.007

Lipids were extracted from the cortex of mice inoculated with fecal microbiota of young mice (+ Microb YM) or old mice (+ Microb OM). The results correspond to quantification of fatty acid methyl esters (FAME) derivatives by GC-FID. The percentage of each fatty acid relative to that of total fatty acids (100%) was determined. Data are expressed as mean ± SEM. * $p < 0.05$ and ** $p < 0.01$.

Supplementary Table 3: Relative amounts of glycerophospholipid species in the cortex.

	**+ Microb YM** (n=7)	**+ Microb OM** (n=8)
**Choline glycerophospholipids (nonhydrolyzed form)**		
Phosphatidylcholine (PC)		
PC (14:0/14:0)*	0.009± 0.000	0.011± 0.001
PC (16:0/16:1)	9.679± 0.155	10.165±0.143
PC (16:0/16:0)	19.640±0.155	19.360±0.183
PC (16:0/17:0)	0.381± 0.007	0.368± 0.006
PC (16:1/18:3)	0.029± 0.001	0.030± 0.001
PC (16:0/18:2)	1.999± 0.034	2.102± 0.069
PC (16:0/18:1)*	22.486±0.080	22.115±0.165
PC (18:0/16:0)	8.403± 0.046	8.359± 0.034
PC (15:0/20:3)	0.006± 0.000	0.006± 0.001
PC (16:1/20:4)	0.377± 0.004	0.409± 0.010
PC (16:0/20:4)	5.036± 0.029	4.852± 0.091
PC (24:3/12:0)	0.326± 0.010	0.311± 0.010
PC (16:0/22:6)	4.391± 0.041	4.435± 0.047
PC (18:1/20:4)	0.704± 0.010	0.687± 0.015
PC (18:0/20:4)	4.333± 0.028	4.211± 0.051
PC (20:4/20:4)	0.271± 0.007	0.279± 0.015
PC (18:1/22:6)	0.487± 0.008	0.487± 0.004
PC (18:0/22:6)	1.865± 0.018	1.942± 0.050
PC (20:1/20:4)	0.124± 0.007	0.107± 0.006
PC (20:0/20:4)	0.445± 0.004	0.435± 0.009

PC (20:1/22:6)	0.040± 0.003	0.047± 0.003
PC (20:0/22:5)	0.035± 0.001	0.036± 0.001
PC (21:0/21:0)*	0.002± 0.000	0.003± 0.000
Plasmenylcholine (PlsC)		
PlsC (18:2p/22:6)	0.471± 0.010	0.510± 0.022
PlsC (18:0p/18:2)	0.041± 0.001	0.045± 0.001
Others		
PC (16:0e/18:2)	0.034± 0.001	0.039± 0.003
PC (16:1e/20:4)	0.024± 0.001	0.023± 0.001
PC (18:0e/18:2)	0.204± 0.008	0.235± 0.013
PC (16:1e/22:6)	0.310± 0.007	0.330± 0.012
Undetermined		
PC (15:0/17:2) / PC (18:1/14:1)	0.060± 0.002	0.068± 0.003
PC (15:0/18:1) / PC (16:0e/18:1)	1.470± 0.017	1.520± 0.019
PC (16:0/18:3) / PC (16:1/18:2)*	0.219± 0.005	0.258± 0.013
PC (15:0/20:4) / PC (16:0e/20:4) / PC (18:2p/18:1)	0.097± 0.002	0.098± 0.001
PC (16:1/19:0) / PC (18:0e/18:1)	0.390± 0.010	0.404± 0.010
PC (18:1/18:1) / PC (18:0/18:2)	3.688± 0.019	3.672± 0.040
PC (18:0/18:1) / PC (16:1p/22:6)	10.542±0.050	10.530±0.063
PC (18:0/19:0) / PC (16:1/22:6) / PC (18:3/20:4)*	0.205± 0.004	0.223± 0.006
PC (16:0/22:1) / PC (18:1p/22:6)	0.952± 0.033	1.072± 0.046
PC (38:3)*	0.212± 0.002	0.202± 0.003
PC (42:6)	0.007± 0.000	0.007± 0.000

PC (42:3)	0.009± 0.001	0.010± 0.001
**Lysophosphatidylcholine (LPC)**		
LPC 16:0	47.481±0.391	47.792±0.337
LPC 18:0	20.109±0.504	21.514±0.431
LPC 18:1	20.403±0.379	19.657±0.420
LPC 18:2	0.698± 0.026	0.691± 0.037
LPC 20:1	0.955± 0.042	1.031± 0.052
LPC 20:2	0.243± 0.006	0.226± 0.006
LPC 20:4	5.637± 0.261	4.778± 0.256
LPC 22:6	4.474± 0.159	4.312± 0.154
**Ethanolamine glycerophospholipids (nonhydrolyzed form)**		
Phosphatidylethanolamine (PE)		
PE (16:0/16:1)	0.127± 0.002	0.134± 0.003
PE (16:0/16:0)	0.199± 0.006	0.197± 0.005
PE (16:0/18:2)*	0.28±6 0.003	0.301± 0.007
PE (16:0/18:1)	5.033± 0.045	5.203± 0.089
PE (16:1/20:4)**	0.217± 0.005	0.252± 0.007
PE (16:0/20:4)	1.745± 0.016	1.701± 0.022
PE (18:1/18:2)	0.337± 0.014	0.312± 0.013
PE (18:0/18:2)	3.476± 0.044	3.689± 0.088
PE (18:0/18:1)	6.196± 0.102	6.434± 0.089
PE (18:3/20:4)**	0.144± 0.004	0.158± 0.004
PE (16:0/22:6)	6.925± 0.064	6.716± 0.093
PE (18:1/20:4)	2.144± 0.098	2.264± 0.018
PE (18:0/20:4)	13.752±0.113	13.150±0.240

PE (20:1/18:2)	0.529± 0.012	0.504± 0.008
PE (20:0/18:2)*	0.714± 0.019	0.812± 0.037
PE (18:2/22:6)	0.149± 0.003	0.157± 0.009
PE (18:1/22:6)	1.883± 0.009	1.871± 0.020
PE (18:0/22:6)*	20.398±0.143	19.765±0.132
PE (18:0/22:4)	5.437± 0.056	5.340± 0.080
PE (16:1/24:2)	0.055± 0.005	0.057± 0.002
PE (18:0/22:1)	0.091± 0.004	0.108± 0.006
PE (20:1/22:6)	0.083± 0.001	0.087± 0.003
PE (20:0/22:6)**	0.048± 0.001	0.044± 0.001
PE (20:0/22:5)	0.087± 0.002	0.087± 0.001
PE (20:0/22:4)	0.143± 0.003	0.138± 0.002
PE (22:6/22:6)	0.207± 0.006	0.246± 0.016
PE (22:5/22:6)	0.020± 0.001	0.021± 0.001
PE (22:4/22:6)	0.147± 0.004	0.149± 0.002
PE (24:6/22:6)	0.006± 0.000	0.007± 0.001
PE (24:4/22:6)	0.012± 0.000	0.011± 0.001
Plasmenylethanolamine (PlsE)		
PlsE (16:0p/16:1)	0.021± 0.001	0.021± 0.001
PlsE (16:0p/16:0)	0.091± 0.002	0.086± 0.002
PlsE (18:0p/14:0)	0.003± 0.000	0.003± 0.000
PlsE (16:0p/18:2)	0.023± 0.001	0.022± 0.001
PlsE (16:0p/18:1)	1.454± 0.038	1.617± 0.056
PlsE (18:0p/16:0)	0.463± 0.007	0.459± 0.005
PlsE (16:0p/20:4)	1.111± 0.025	1.098± 0.024

PlsE (16:0p/20:3)	0.070± 0.001	0.075± 0.002
PlsE (18:1p/18:2)	0.030± 0.001	0.030± 0.002
PlsE (18:0p/18:2)	0.042± 0.002	0.044± 0.002
PlsE (18:1p/18:0)*	0.027± 0.001	0.031± 0.000
PlsE (18:0p/18:1)	2.700± 0.063	2.870± 0.063
PlsE (16:0p/20:0)**	0.011± 0.001	0.015± 0.001
PlsE (18:0p/18:0)	0.009± 0.000	0.010± 0.001
PlsE (16:0p/22:6)	3.260± 0.026	3.173± 0.041
PlsE (18:1p/20:4)	1.031± 0.009	1.042± 0.018
PlsE (16:0p/22:4)	0.812± 0.014	0.788± 0.013
PlsE (18:1p/20:3)	0.497± 0.007	0.499± 0.010
PlsE (18:0p/20:4)	2.418± 0.019	2.391± 0.024
PlsE (16:0p/22:3)	0.083± 0.003	0.091± 0.003
PlsE (18:1p/20:2)	0.117± 0.003	0.129± 0.005
PlsE (18:0p/20:3)	0.225± 0.004	0.242± 0.007
PlsE (16:0p/22:2)	0.010± 0.000	0.012± 0.001
PlsE (18:1p/20:1)*	1.492± 0.044	1.678± 0.056
PlsE (18:0p/20:2)	0.095± 0.002	0.102± 0.004
PlsE (16:0p/22:1)	0.024± 0.001	0.027± 0.001
PlsE (18:1p/20:0)*	0.113± 0.006	0.145± 0.008
PlsE (18:0p/20:1)	1.192± 0.040	1.350± 0.049
PlsE (18:0p/20:0)*	0.038± 0.003	0.050± 0.004
PlsE (18:1p/22:6)	1.251± 0.010	1.238± 0.015
PlsE (18:0p/22:6)	4.846± 0.051	4.713± 0.041
PlsE (18:1p/22:4)	0.888± 0.021	0.931± 0.020

PlsE (16:0p/24:4)	0.037± 0.001	0.035± 0.001
PlsE (18:1p/22:3)	0.763± 0.013	0.729± 0.018
PlsE (18:0p/22:4)	1.666± 0.023	1.644± 0.027
PlsE (18:1p/22:2)	0.037± 0.001	0.041± 0.002
PlsE (18:0p/22:3)	0.093± 0.003	0.105± 0.004
PlsE (18:1p/22:1)*	0.122± 0.006	0.145± 0.005
PlsE (18:0p/22:2)	0.023± 0.001	0.026± 0.002
PlsE (18:0p/22:1)**	0.087± 0.003	0.103± 0.005
PlsE (20:1p/22:6)	0.030± 0.001	0.030± 0.001
PlsE (20:0p/22:6)	0.086± 0.003	0.083± 0.003
Undetermined		
PE (16:0/18:0) / PE (17:0/17:0)	0.670± 0.013	0.667± 0.012
PE 36:0	0.045± 0.003	0.043± 0.003
PE 38:1*	0.599± 0.017	0.688± 0.026
PE 40:2 *	0.131± 0.005	0.163± 0.011
PE 40:5	0.450± 0.011	0.458± 0.019
PE 42:1*	0.035± 0.003	0.049± 0.004
PE 42:2 **	0.030± 0.002	0.039± 0.002
PE 42:8	0.057± 0.002	0.055± 0.001
**Lysophosphatidylethanolamine (LPE)**		
LPE 18:0*	12.160±0.262	13.137±0.202
LPE 18:1	20.907±0.638	22.261±0.681
LPE 18:2	0.367± 0.013	0.351± 0.013
LPE 20:4*	17.629±0.160	16.888±0.262
LPE 22:6*	48.937±0.423	47.363±0.465

**Inositol glycerophospholipids (nonhydrolyzed form)**		
Phosphatidylinositol (PI)		
PI (16:0/18:2)	0.165± 0.015	0.179± 0.011
PI (16:0/18:1)	2.183± 0.102	2.405± 0.088
PI (18:0/16:0)	1.121± 0.081	1.248± 0.035
PI (16:0/20:4)	9.776± 0.318	9.344± 0.176
PI (18:1/18:2)	0.145± 0.007	0.162± 0.009
PI (18:1/18:1)	0.617± 0.015	0.665± 0.033
PI (18:0/18:1)	2.157± 0.097	2.286± 0.054
PI (20:0/16:0)	0.041± 0.006	0.043± 0.005
PI (17:0/20:4)	0.412± 0.025	0.433± 0.010
PI (16:0/22:6)	1.277± 0.049	1.339± 0.055
PI (18:0/20:4)	61.556±0.346	61.653±0.187
PI (18:0/22:6)	1.893± 0.056	2.118± 0.114
Plasmenylinositol (PlsI)		
PlsI (18:0p/20:4)	11.215±0.226	10.270±0.409
Undetermined		
PI (18:0/20:5) / PI (18:1/20:4)	7.443± 0.054	7.855± 0.159

Lipids were extracted from the cortex of mice inoculated with gut microbiota of young mice (+ Microb YM) or old mice (+ Microb OM). Lipid species were analyzed by LC-MS^2. For each glycerophospholipid class, results are expressed as abundance (in percentage) of each species relative to that of total species, defined as 100%. Data are expressed as mean ± SEM. * $p < 0.05$ and ** $p < 0.01$.

Supplementary Table 4: Relative levels of sphingomyelin species in the cortex.

**Sphingomyelin species**	**+ Microb YM** (n=7)	**+ Microb OM** (n=8)
SM (d18:1/16:0)	6.373± 0.240	6.596± 0.327
SM (d16:1/17:0)	0.053± 0.003	0.057± 0.004
SM (d18:1/16:1)	0.158± 0.007	0.175± 0.010
SM (d18:0/16:0) *	0.906± 0.017	1.005± 0.026
SM (d18:1/17:0) *	1.006± 0.018	0.946± 0.017
SM (d18:2/18:0)	20.140±0.418	19.686±0.573
SM (d18:1/18:0)*	50.830±0.485	49.248±0.674
SM (d18:0/18:0)	0.714± 0.045	0.624± 0.030
SM (d18:2/20:0)	0.154± 0.006	0.163± 0.004
SM (d18:2/22:0)	0.485± 0.011	0.524± 0.018
SM (d18:1/22:0)	2.247± 0.066	2.491± 0.147
SM (d18:2/24:1)	0.339± 0.014	0.360± 0.015
SM (d18:1/24:1) *	8.709± 0.242	9.894± 0.425
SM (d18:1/24:0)	1.751± 0.055	2.025± 0.121
Undetermined		
SM (d18:1/20:0) / SM (d20:0/18:1)	6.135± 0.153	6.207± 0.149

Lipids were extracted from the cortex of mice inoculated with gut microbiota of young mice (+ Microb YM) or old mice (+ Microb OM). Sphingomyelin species were analyzed by LC-MS^2. Results are expressed as abundance (in percentage) of each species relative to that of total species, defined as 100%. Data are expressed as mean ± SEM. * $p < 0.05$.
